# First Isolation of Severe Fever with Thrombocytopenia Syndrome Virus from *Haemaphysalis longicornis* Ticks Collected in Severe Fever with Thrombocytopenia Syndrome Outbreak Areas in the Republic of Korea

**DOI:** 10.1089/vbz.2015.1832

**Published:** 2016-01-01

**Authors:** Seok-Min Yun, Bong Gu Song, WooYoung Choi, Jong Yul Roh, Ye-Ji Lee, Won Il Park, Myung Guk Han, Young Ran Ju, Won-Ja Lee

**Affiliations:** ^1^ Division of Arboviruses, National Institute of Health, Korea Centers for Disease Control and Prevention, Cheongju-si, Republic of Korea.; ^2^ Division of Medical Entomology, National Institute of Health, Korea Centers for Disease Control and Prevention, Cheongju-si, Republic of Korea.

**Keywords:** SFTSV, Isolate, *Haemaphysalis longicornis*, Republic of Korea

## Abstract

Severe fever with thrombocytopenia syndrome (SFTS) is an emerging tick-borne infectious disease that is endemic to China, Japan, and the Republic of Korea (ROK). In this study, 8313 ticks collected from SFTS outbreak areas in the ROK in 2013 were used to detect the SFTS virus (SFTSV). A single SFTSV was isolated in cell culture from one pool of *Haemaphysalis longicornis* ticks collected from Samcheok-si, Gangwon Province, in the ROK. Phylogenetic analysis showed that the SFTSV isolate was clustered with the SFTSV strain from Japan, which was isolated from humans. To the best of our knowledge, this is the first isolation in the world of SFTSV in ticks collected from vegetation.

## Introduction

Severe fever with thrombocytopenia syndrome (SFTS) is an emerging tick-borne viral disease characterized by fever, gastrointestinal symptoms, leukopenia, and thrombocytopenia (Yu et al. 2011). The causative agent of the disease is the SFTS virus (SFTSV), a recently identified phlebovirus in the family Bunyaviridae (Yu et al. 2011). The genome of SFTSV consists of three single-stranded, negative-sense RNA segments, designated large (L), medium (M), and small (S) (Yu et al. 2011). SFTS cases were reported for the first time in China (Yu et al. 2011) in 2010, and in the Republic of Korea (ROK) (Kim et al. 2013, Park et al. 2014a) and Japan (Takahashi et al. 2014) in 2013. Other novel tick-borne phleboviruses, Heartland virus (HRTV) and Hunter Island Group virus (HIGV), which are genetically related to but distinctly different from SFTSV, have been isolated from leukocytes of patients in the United States (McMullan et al. 2012) and ticks in Australia (Wang et al. 2014), respectively.

Although human-to-human transmission of SFTSV through contact with an infected patient's blood or mucus has been reported (Liu et al. 2012, Gai et al. 2012, Kim et al. 2015), the virus is transmitted to humans predominantly by tick bites. The SFTSV has been previously detected in tick species, including *Haemaphysalis longicornis* and *Rhipicephalus microplus* (Yu et al. 2011, Zhang et al. 2012). In our study on SFTSV in ticks collected from humans in the ROK, *Amblyomma testudinarium* and *Ixodes nipponensis* ticks were also implicated as potential SFTSV vectors (Yun et al. 2014). Previous studies regarding SFTSV show that domestic animals, such as goats, cattle, dogs, and chickens, and small mammals, such as rodents and shrews, can act as hosts of SFTSV (Jiao et al. 2012, Zhao et al. 2012, Niu et al. 2013, Cui et al. 2013, Liu et al. 2014).

In the ROK, an SFTS case had not been reported prior to a fatal case in 2012 that was identified in Gangwon Province (Kim et al. 2013). Since then, a total of 36 cases, with a mortality rate of 47.2%, has been reported in several regions in the ROK, and 26 virus isolates were obtained from the serum of SFTS patients in 2013 (Park et al. 2014a). Our previous studies reported the existence and prevalence of SFTSV in ticks from the ROK (Yun et al. 2014, Park et al. 2014b). These results showed evidence that SFTS is endemic to several regions of the ROK. However, no studies have focused on the detection of SFTSV in ticks collected from areas within the ROK in which human cases have occurred. To verify the correlation between SFTSV prevalence in ticks and SFTS outbreaks and to determine the possible vector of SFTSV, we aimed to investigate the prevalence of SFTSV in ticks collected from SFTS outbreak areas in the ROK in 2013 and attempted to isolate SFTSV from positive tick pools.

## Materials and Methods

**FIG. 1.**
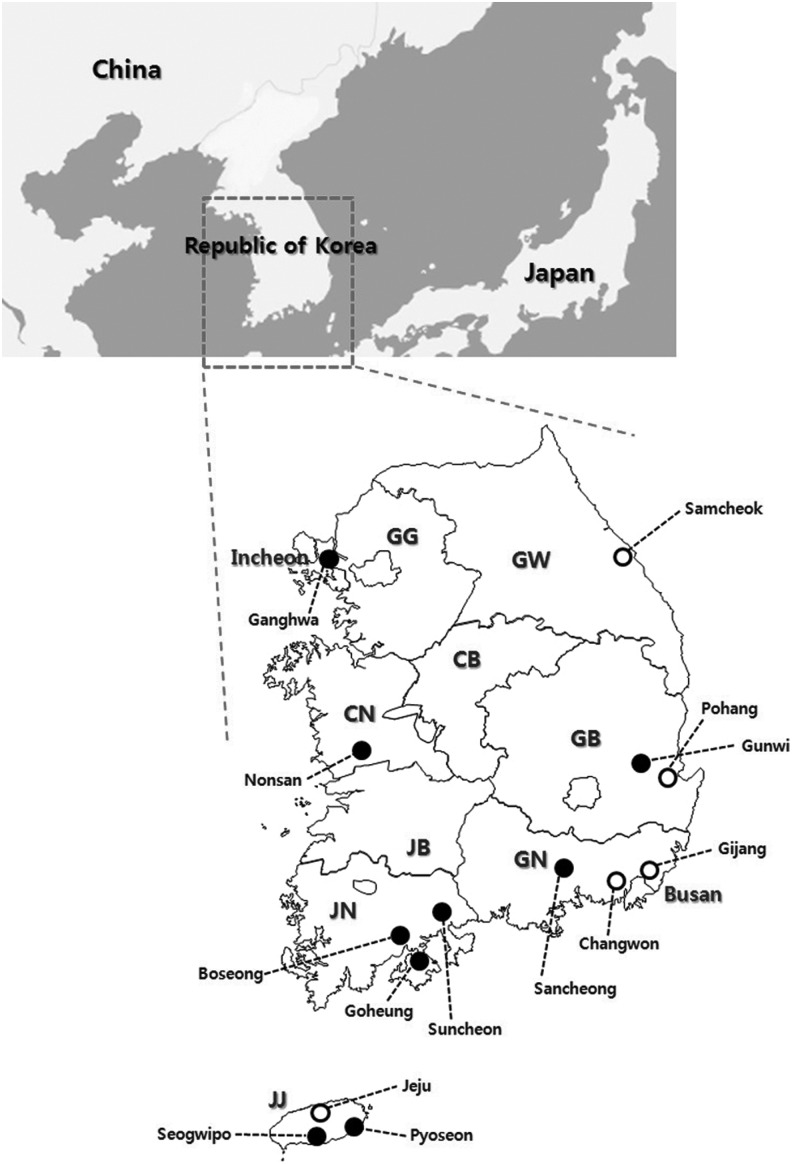
Geographical location of collection sites in this study. Open circles show the sites in which positive tick pools for SFTSV were detected. GG, Gyeonggi Province; GW, Gangwon Province; CB, Chungcheongbuk Province; CN, Chungcheongnam Province; JB, Jeonllabuk Province; JN, Jeonllanam Province; GB, Gyeongsangbuk Province; GN, Gyeongsangnam Province; JJ, Jeju Special Autonomous Province.

Ticks were collected by the flagging and dragging method or by using dry ice–baited tick traps at 14 sites from vegetation in six provinces and two metropolitan cities in the ROK in 2013 (Fig. 1). These sites were selected for the survey based on the number of SFTS cases identified in humans in these regions. Ticks were collected at or near the residences of human cases of SFTS. After collection, the ticks were placed in plastic tubes and transported to the laboratory to identify the species and developmental stage under a dissecting microscope according to a reported classification method (Yamaguti et al. 1971).

Tick samples were pooled according to species, developmental stage, and site (1–50 larvae per pool, 1–30 nymphs per pool, and 1–5 adults per pool). The pooled ticks were homogenized in 600 μL of phosphate-buffered saline (PBS; pH 7.0) containing 10% fetal bovine serum (FBS; Gibco BRL, Grand Island, NY), penicillin (500 IU/mL, Gibco BRL), and streptomycin (500 μg/mL, Gibco BRL) using a Precellys^®^ 24 homogenizer (Bertin Technologies, Bretonneux, France) and 2.8-mm stainless steel beads. The tick homogenates were centrifuged at 8600 × *g* for 5 min, and RNA was extracted from the supernatants of the tick homogenates according to the manufacturer's instructions using a Viral Gene-spin^™^ RNA Extraction Kit (iNtRON Biotechnology, Seongnam, ROK). To detect the SFTSV M segment gene in tick homogenates, one-step reverse transcription polymerase chain reaction (RT-PCR) was conducted using a DiaStar 2X OneStep RT-PCR Premix Kit (SolGent, Daejeon, ROK) via a previously described method (Yun et al. 2014). The RT-PCR amplicon was purified using a QIAquick^®^ Gel Extraction Kit (QIAGEN, Valencia, CA) according to the manufacturer's instructions and sequenced after cloning into a pCR^®^2.1-TOPO^®^ plasmid (Invitrogen, Carlsbad, CA) using an ABI Prism BigDye Terminator Cycle Sequencing Kit and an ABI 3730xl Sequencer (Applied Biosystems, Foster City, CA) at Macrogen Inc. (Daejeon, ROK).

To isolate SFTSV from positive tick pools, tick homogenates were added to an equal volume of Dulbecco's Modified Eagle Medium (DMEM; Gibco BRL) containing penicillin (5000 IU/mL, Gibco BRL), and streptomycin (5 mg/mL, Gibco BRL). The mixture was filtered using a 0.45-μm syringe filter and clarified by centrifugation, and the supernatants were used as the inoculums for virus isolation. Vero E6 cell monolayers formed in 12-well plates were inoculated with the supernatants and incubated at 37°C for 7 days. Then, cell suspensions were passaged to new monolayers of Vero E6 cells, as previously described (Park et al. 2014a), and stored at −70°C until use.

To identify the isolated virus, we examined the presence of the SFTSV M segment gene in passaged cell supernatants using RT-PCR and confirmed the results by indirect immunofluorescent antibody assay (IFA), immunoblotting using a monoclonal SFTSV nucleocapsid (N) protein antibody (manufactured by our laboratory), and transmission electron microscopy (TEM) analysis for morphological identification.

For phylogenetic analysis, sequence alignment and construction of the phylogenetic tree were performed using MEGA software version 6.0 (Tamura et al. 2013).

## Results and Discussion

A total of 8313 ticks belonging to three genera and four species (*H. longicornis*, *Haemaphysalis flava*, *I. nipponensis*, and *A. testudinarium*) were collected. Of the identified ticks, *H. longicornis* (*n* = 8230; 99%) was the most frequently collected species in this study, followed by *H. flava* (*n* = 73; 0.88%), *I. nipponensis* (*n* = 9; 0.11%), and *A. testudinarium* (*n* = 1; 0.01%). All ticks collected in this study had not fed. Among them, SFTSV RNA was detected in nine pools (eight pools in *H. longicornis* and one pool in *H. flava*) (Table 1). The SFTSV minimum infection rate per 100 ticks (minimum infection rate [MIR] = no. of positive pools/no. of examined ticks in pools ×100) was 0.11% (nine pools/8313 individuals). These results suggest that *H. longicornis* and *H. flava*, from which SFTSV genome was detected, serve as potential vectors in the ROK, although *H. flava* has not been previously considered as a SFTSV vector. Additionally, SFTSV RNA was detected in *H. longicornis* larva, suggesting that SFTSV may possibly be transmitted by the transovarial route. To support this hypothesis, SFTSV will need to be isolated from infected ticks (potential vectors) and the virus characterized further. Laboratory vector competence studies are needed to confirm the transovarial transmission of SFTSV in ticks.

**Table 1.** Information on Tick Pools Positive for Severe Fever with Thrombocytopenia Syndrome Virus RNA in This Study

*Collection sites*					
*Province or Metropolitan city*	*Regions*	*Species*	*No. ticks (L/N/M/F)*^a^	*Tick pools positive by RT-PCR (MIR,*^b^*developmental stage)*	*Tick pool positive by virus isolation (strain)*
Jeju Special Autonomous Province	Jeju-si	*Haemaphysalis longicornis*	679 (0/582/36/61)	1 (0.15%, nymph)	^−c^
Gangwon Province	Samcheok-si	*Haemaphysalis longicornis*	640 (32/530/15/63)	3 (0.47%, nymph)	1 (KAGWT strain)
Busan Metropolitan city	Gijang-gun	*Haemaphysalis longicornis*	2 (1/0/1/0)	1 (50%, larva)	^−^
		*Haemaphysalis flava*	663 (654/9/0/0)	1 (0.15%, adult male)	^−^
Gyeongsangbuk Province	Pohang-si	*Haemaphysalis longicornis*	887 (123/492/37/235)	1 (0.11%, adult female)	^−^
Gyeongsangnam Province	Changwon-si	*Haemaphysalis longicornis*	156 (94/60/0/2)	2 (1.29%, nymph)	^−^

^a^ L, larvae; N, nymphs; M, adult males; F, adult females.

^b^ MIR = Minimum infection rate per 100 ticks (no. of positive pools/no. of examined ticks in pools ×100).

^c^ −, Negative result in the virus isolation test.

**FIG. 2.**
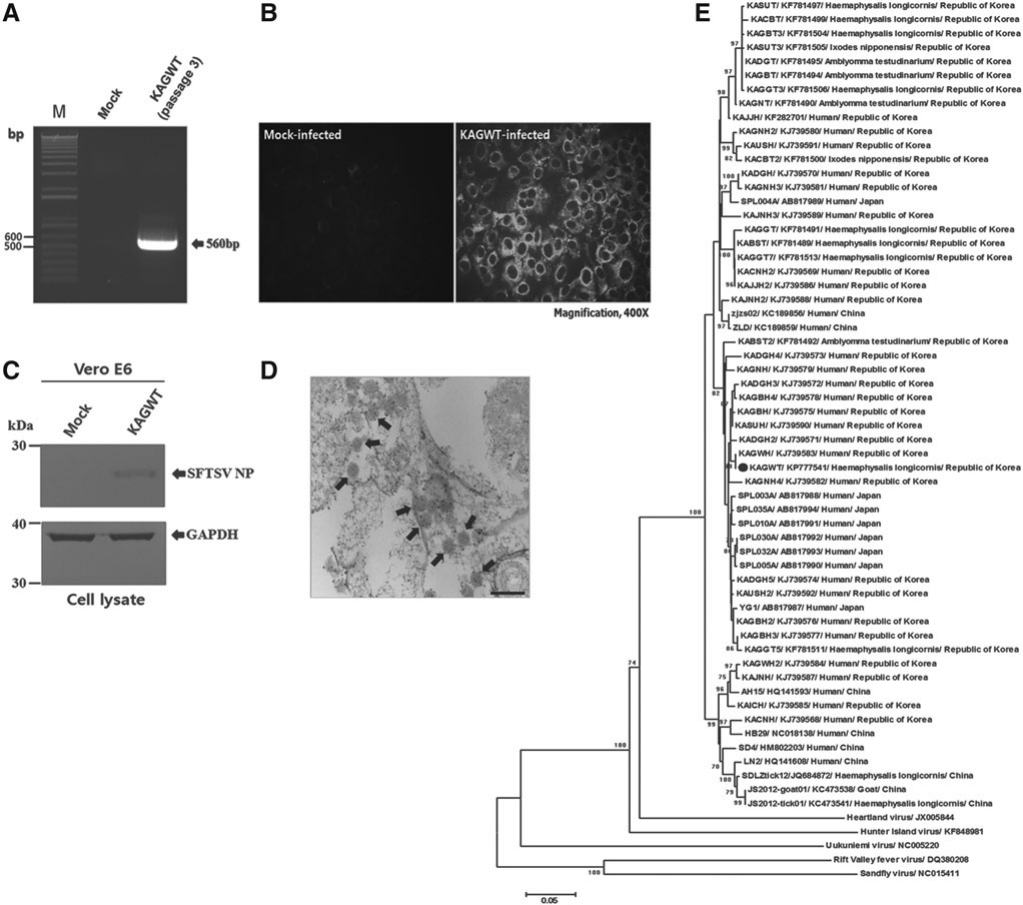
Identification of severe fever with thrombocytopenia syndrome virus (SFTSV) isolated from ticks and phylogenetic analysis of SFTSV strains. (**A**) Detection of SFTSV M segment gene (560 bp) from the supernatant of the KAGWT-infected cell culture after three passages by RT-PCR. Lane M, 1-kb DNA plus ladder. Identification of antigenicity by indirect immunofluorescent assay (IFA) (**B**) and immunoblotting (**C**) for Vero E6 cells infected with KAGWT using anti-SFTSV nucleoprotein (NP) monoclonal antibody. Glyceraldehyde 3-phosphate dehydrogenase (GAPDH) was used as a protein loading control. (**D**) Transmission electron microscopy (TEM) image of Vero E6 cells infected with the KAGWT strain (arrows). Scale bar in the image indicates 200 nm. Magnification, 80,000×. (**E**) Phylogenetic analysis of SFTSV strains based on the partial M segment sequences. Phylogenetic trees constructed using the neighbor-joining (NJ) method based on the *p*-distance model in MEGA version 6 (5000 bootstrap replicates). The phylogenetic branches were supported with greater than 70% bootstrap values in this analysis. Uukuniemi virus, Rift Valley fever virus, Heartland virus, Hunter Island Group virus, and Sandfly virus were used as outgroups. The scale bar indicates the nucleotide substitutions per position. Each strain is identified by strain name followed by GenBank accession number, source of virus, and geographical origin, except for the five outgroups. The Korean strain isolated in this study is marked with a closed circle.

Of the nine SFTSV-positive tick pools, only one pool of *H. longicornis* nymphs collected from Samcheok-si, Gangwon Province, was positive in the virus isolation test. As shown in Figure 2, the virus isolate designated as KAGWT from *H. longicornis* was identified as a SFTSV. The sequence of KAGWT determined in this study has been deposited in GenBank (acc. no. KP777541).

The partial M segment genome-based phylogenetic analysis revealed that the SFTSV KAGWT was more closely related to the SFTSV strain from Japan, which was isolated from humans. Our result is consistent with a previous report (Park et al. 2014a) indicating that SFTSV Korean isolates from humans are closely related to Japanese isolates. To our knowledge, this is the first survey of SFTSV prevalence in ticks collected from SFTS outbreak areas of the ROK, and this article describes studies that identify the first isolation of SFTSV from ticks collected in the ROK.

In conclusion, it was demonstrated that SFTSV-infected ticks were distributed in several areas of the ROK in which an SFTS outbreaks occurred in 2013. Furthermore, SFTSV was first isolated from *H. longicornis* ticks collected in the study area in which the SFTS human case occurred. This suggests that *H. longicornis* is a vector for SFTSV in the ROK. Although virus isolation from *H. flava* failed, we suspect that this tick may play a role in SFTSV transmission. These findings emphasize the need for continuous tick-based surveillance for SFTSV to monitor information about SFTSV activity and distribution in the ROK. Taken together, further research, such as whole genome analysis of a tick-derived SFTSV Korean isolate, is needed to understand better the genetic diversity and molecular evolution of SFTSV.

## References

[B1] Cui F, Cao HX, Wang L, Zhang SF, et al. Clinical and epidemiological study on severe fever with thrombocytopenia syndrome in Yiyuan County, Shandong Province, China. Am J Trop Med Hyg 2013; 88:510–512 23339197 10.4269/ajtmh.11-0760 PMC3592533

[B2] Gai Z, Liang M, Zhang Y, Zhang S, et al. Person-to-person transmission of severe fever with thrombocytopenia syndrome bunyavirus through blood contact. Clin Infect Dis 2012; 54:249–252 22095565 10.1093/cid/cir776 PMC3245727

[B3] Jiao Y, Zeng X, Guo X, Qi X, et al. Preparation and evaluation of recombinant severe fever with thrombocytopenia syndrome virus nucleocapsid protein for detection of total antibodies in human and animal sera by double-antigen sandwich enzyme-linked immunosorbent assay. J Clin Microbiol 2012; 50:372–377 22135253 10.1128/JCM.01319-11 PMC3264160

[B4] Kim KH, Yi J, Kim G, Choi SJ, et al. Severe fever with thrombocytopenia syndrome, South Korea, 2012. Emerg Infect Dis 2013; 19:1892–1894 24206586 10.3201/eid1911.130792 PMC3837670

[B5] Kim WY, Choi W, Park SW, Wang EB, et al. Nosocomial transmission of severe fever with thrombocytopenia syndrome in Korea. Clin Infect Dis 2015; 60:1681–1683 25694652 10.1093/cid/civ128

[B6] Liu JW, Wen HL, Fang LZ, Zhang ZT, et al. Prevalence of SFTSV among Asian house shrews and rodents, China, January–August 2013. Emerg Infect Dis 2014; 20:2126–2128 25418111 10.3201/eid2012.141013 PMC4257798

[B7] Liu Y, Li Q, Hu W, Wu J, et al. Person-to-person transmission of severe fever with thrombocytopenia syndrome virus. Vector Borne Zoonotic Dis 2012; 12:156–160 21955213 10.1089/vbz.2011.0758

[B8] McMullan LK, Folk SM, Kelly AJ, MacNeil A, et al. A new phlebovirus associated with severe febrile illness in Missouri. N Engl J Med 2012; 367:834–841 22931317 10.1056/NEJMoa1203378

[B9] Niu G, Li J, Liang M, Jiang X, et al. Severe fever with thrombocytopenia syndrome virus among domesticated animals, China. Emerg Infect Dis 2013; 19:756–763 23648209 10.3201/eid1905.120245 PMC3647489

[B10] Park SW, Han MG, Yun SM, Park C, et al. Severe fever with thrombocytopenia syndrome virus, South Korea, 2013. Emerg Infect Dis 2014a; 20:1880–1882 25341085 10.3201/eid2011.140888 PMC4214315

[B11] Park SW, Song BG, Shin EH, Yun SM, et al. Prevalence of severe fever with thrombocytopenia syndrome virus in *Haemaphysalis longicornis* ticks in South Korea. Ticks Tick Borne Dis 2014b; 5:975–977 25164614 10.1016/j.ttbdis.2014.07.020

[B12] Takahashi T, Maeda K, Suzuki T, Ishido A, et al. The first identification and retrospective study of severe fever with thrombocytopenia syndrome in Japan. J Infect Dis 2014; 209:816–827 24231186 10.1093/infdis/jit603 PMC7107388

[B13] Tamura K, Stecher G, Peterson D, Filipski A, et al. MEGA6: Molecular Evolutionary Genetics Analysis version 6.0. Mol Biol Evol 2013; 30:2725–2729 24132122 10.1093/molbev/mst197 PMC3840312

[B14] Wang J, Selleck P, Yu M, Ha W, et al. Novel phlebovirus with zoonotic potential isolated from ticks, Australia. Emerg Infect Dis 2014; 20:1040–1043 24856477 10.3201/eid2006.140003 PMC4036776

[B15] Yamaguti N, Tipton VJ, Keegan HL, Toshioka S Ticks of Japan, Korea, and the Ryukyu Islands. Brigham Young University Science Bulletin, 1971; 15:1–226

[B16] Yu XJ, Liang MF, Zhang SY, Liu Y, et al. Fever with thrombocytopenia associated with a novel bunyavirus in China. N Engl J Med 2011; 364:1523–1532 21410387 10.1056/NEJMoa1010095 PMC3113718

[B17] Yun SM, Lee WG, Ryou J, Yang SC, et al. Severe fever with thrombocytopenia syndrome virus in ticks collected from humans, South Korea, 2013. Emerg Infect Dis 2014; 20:1358–1361 25061851 10.3201/eid2008.131857 PMC4111194

[B18] Zhang YZ, Zhou DJ, Qin XC, Tian JH, et al. The ecology, genetic diversity, and phylogeny of Huaiyangshan virus in China. J Virol 2012; 86:2864–2868 22190717 10.1128/JVI.06192-11 PMC3302241

[B19] Zhao L, Zhai S, Wen H, Cui F, et al. Severe fever with thrombocytopenia syndrome virus, Shandong Province, China. Emerg Infect Dis 2012; 18:963–965 22608264 10.3201/eid1806.111345 PMC3358154

